# The emerging role of radical chemistry in the amination transformation of highly strained [1.1.1]propellane: Bicyclo[1.1.1]pentylamine as bioisosteres of anilines

**DOI:** 10.3389/fchem.2022.997944

**Published:** 2022-10-21

**Authors:** Qiwen Pang, Yang Li, Xin Xie, Jie Tang, Qian Liu, Cheng Peng, Xiang Li, Bo Han

**Affiliations:** State Key Laboratory of Southwestern Chinese Medicine Resources, Hospital of Chengdu University of Traditional Chinese Medicine, School of Pharmacy and College of Medical Technology, Chengdu University of Traditional Chinese Medicine, Chengdu, China

**Keywords:** [1.1.1]propellane, bicyclo[1.1.1]pentylamine, radical chemistry, amination, bioisosteres

## Abstract

Bicyclo[1.1.1]pentylamines (BPCAs), emerging as sp^3^-rich surrogates for aniline and its derivatives, demonstrate unique structural features and physicochemical profiles in medicinal and synthetic chemistry. In recent years, compared with conventional synthetic approaches, the rapid development of radical chemistry enables the assembly of valuable bicyclo[1.1.1]pentylamines scaffold directly through the amination transformation of highly strained [1.1.1]propellane. In this review, we concisely summarize the emerging role of radical chemistry in the construction of BCPAs motif, highlighting two different and powerful radical-involved strategies including *C*-centered and *N*-centered radical pathways under appropriate conditions. The future direction concerning BCPAs is also discussed at the end of this review, which aims to provide some inspiration for the research of this promising project.

## Introduction

**Scheme 1:**
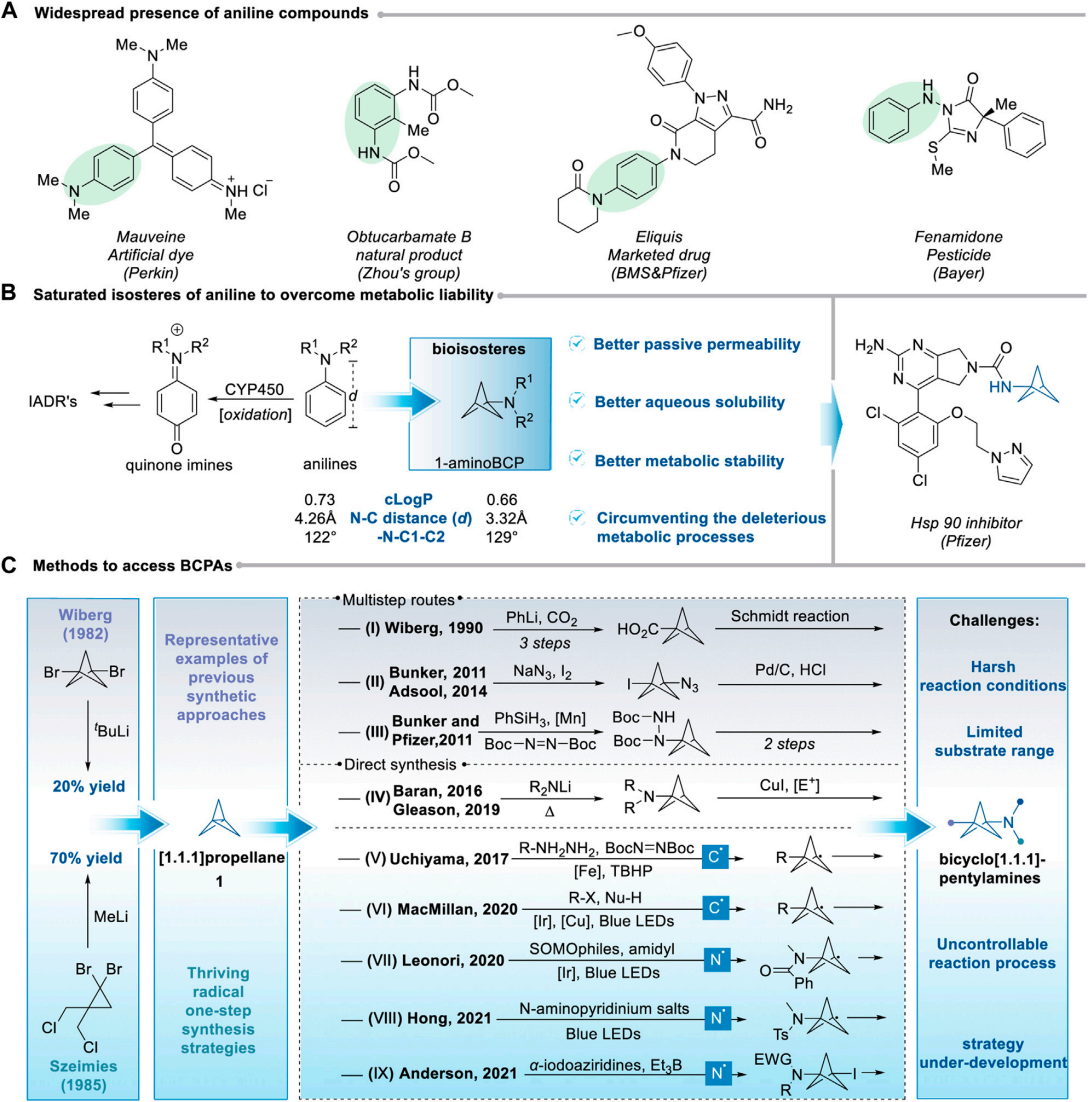
Advancements of BCPAs in the medicinal and synthetic chemistry.

As one of the primary and versatile materials, aniline moved to the center of the stage in the history of the chemical industry in 1856 due to the synthesis of the first artificial dye called mauveine by British chemist *William Henry Perkin* (Scheme 1A, left) (Perkin, 1896). Despite the significance of aniline utilized as an essential chemical in the dye industry and industrial production of rubber chemicals and 4,4′-diphenylmethane diisocyanate (MDI) (North, 2005), the remarkable progress has also been witnessed in the discovery of novel medicine and powerful pesticide. As a valuable and common structural element, aniline and its derivatives widely exist in various bioactive natural products, pharmaceuticals, clinical drugs, and pesticides (Scheme 1A, right) (Lacroix et al., 1999; Gan et al., 2013; Greig and Garnock-Jones, 2016; Desideri et al., 2019). According to the statistics supported by the University of Arizona, approximately 54% and 65% of top 200 small molecule pharmaceuticals by retail sales in 2020 and 2021, respectively, contain at least an aniline unit (McGrath et al., 2010).

With the rise of green chemistry and precision therapy, the pursuit of targeted drugs featuring excellent tissue and cell selectivity (Wang et al., 2020; Chu et al., 2021; Sun et al., 2021), high efficiency, and the environment-friendly synthetic procedure (Peng et al., 2019; Zuo et al., 2021) has increasingly become a research trend in medicinal (Friedman et al., 2015; Soto et al., 2020; Manzari et al., 2021; Mitchell et al., 2021) and synthetic chemistry (Dunn, 2012; Simon and Li, 2012; Galuszka et al., 2013; Newman and Jensen, 2013; Jahangirian et al., 2017; Rogers and Jensen, 2019). These concepts put higher requirements on the metabolic tolerance (Arnott and Planey, 2012; Wishart, 2016; Quinn et al., 2017; Yang et al., 2019; Bi et al., 2020), pharmacokinetic properties (Ferreira and Andricopulo, 2019; Yang et al., 2019; Jia et al., 2020), and synthetic process of new drugs (Nadin et al., 2012; Gerry and Schreiber, 2018; Campos et al., 2019; Lenci and Trabocchi, 2020; Han et al., 2021). Although aniline and its derivatives have become a prevalent substructure in discovering new drugs, their propensity for reactive metabolite (RM) formation triggered by cytochrome P450 (CYP450)-mediated oxidation may cause drug−drug interactions or adverse drug interactions (Scheme 1B, left) (Stepan et al., 2011; Orr et al., 2012; Kalgutkar, 2015). As a result, in some cases, aniline unit(s) incorporated into the structure of drug candidates cannot fulfill the high standard mentioned above. One of the most typical cases is the structural optimization of the Hsp 90 inhibitor due to the resistance towards metabolic clearance caused by benzamide moiety (Scheme 1B, right) (Zehnder et al., 2008; Zehnder et al., 2011). Hence, developing a strategic replacement of the aniline unit(s) to address these challenges is highly demanded.

In modern drug discovery, installing a three-dimensional small-ring framework into the structure of drug candidates provides unique opportunities to expand potential drug-like chemical space and alert the physicochemical profiles while maintaining comparable levels of bioactivity (Carreira and Fessard, 2014; Auberson et al., 2017; Measom et al., 2017). The bicyclo[1.1.1]pentanes (BCPs) have recently received considerable attention from the pharmaceutical, agrochemical, and materials industries. It has have become established as useful sp^3^-rich surrogates for arenes (Stepan et al., 2012; Mykhailiuk, 2019), tert-butyl groups (Westphal et al., 2015), and alkynes functional groups (Makarov et al., 2017). As the potential nontoxic bioisosteres for aniline and *N*-tert-butyl moieties, bicyclo[1.1.1]pentylamines (BCPAs) offer straightforward access to improve medicinal properties in drug candidates (Lovering et al., 2009). In particularly, the BCPAs motif demonstrates significant interest in circumventing the deleterious metabolic processes of an aniline moiety because of its higher Fsp3 and three-dimensionality, and its spatial approximation to aniline in physical parameters (Scheme 1B) (Walsh and Miwa, 2011; Kalgutkar, 2015; Sodano et al., 2020). Indeed, this strategic replacement has been successfully applied to optimize the metabolic level of the Hsp 90 inhibitor developed by Pfizer Company (Scheme 1B, right) (Zehnder et al., 2008; Zehnder et al., 2011). Recognizing the critical value of BCPAs scaffold, tremendous efforts from the scientific community have been increasingly devoted to efficiently constructing BCPAs scaffold over the past decades (Sodano et al., 2020).

From the retrosynthesis point of view, BCPAs can be smoothly and (in)directly produced by amination functionalization of the highly strained [1.1.1]propellane at the bridgehead position (Wiberg and Waddell, 1990; Milligan and Wipf, 2016; Sterling et al., 2020), where the internal central C-C bond can be readily cleaved under a strain-release approach (Gianatassio et al., 2016; Lopchuk et al., 2017). And the [1.1.1]propellane can be easily obtained by intramolecular reductive coupling reaction of 1,3-dibromobicyclo[1.1.1]pentane firstly reported by Wiberg and Walker in 1982, and later by using 1,1-dibromo-2,2-bis(chloromethyl)cyclopropane as starting material developed by Szeimies and co-workers (Scheme 1C, left) (Wiberg and Walker, 1982; Semmler et al., 1985). Early approaches to BCPAs involved multistep routes utilizing preformed BCP building blocks derived from a strained [1.1.1]propellane, such as acyl nitrene rearrangements or reduction of BCP azides or hydrazines (Scheme 1C, I–III) (Wiberg and Waddell, 1990; Bunker et al., 2011; Goh et al., 2014). In 2006, the breakthrough was made by Baran et al., who developed the strain-release amination of [1.1.1]propellane leading to access to monosubstituted BCPAs (Scheme 1C, IV) (Gianatassio et al., 2016; Lopchuk et al., 2017). Then, the Gleason group extended this approach to synthesizing 1,3-disubstituted BCPAs (Scheme 1C, IV) (Hughes et al., 2019). However, these processes require elevated temperatures, restricting functional group tolerance.

In recent years, fast and impressive achievements have been witnessed in radical chemistry, and several approaches for constructing BCPAs have been continuously established (Chen et al., 2016; Oberg, 2016; Studer and Curran, 2016; Yu et al., 2020; Parsaee et al., 2021; Sumida and Ohmiya, 2021; Chan et al., 2022; Holmberg-Douglas and Nicewicz, 2022). On the one hand, adding *C*-centered radicals [generated under Fe(II) or metalaphotoredox catalysis] to [1.1.1]propellane offers alternative solutions for synthesizing BCPAs in a rapid and reaction manner (Scheme 1C, V–VI) (Kanazawa et al., 2017; Zhang et al., 2020). On the other hand, in contrast to the amination of BCPAs formed by the addition of *C*-centered radicals, reactions with the direct addition of *N*-centered radicals to [1.1.1]propellane seem like a concise and promising approach to BCPAs frameworks. However, this strategy lay dormant for more than 30 years after the seminal work of Wiberg on the reaction of BCP with nitric oxide (Wiberg and Waddell, 1990). At present, the addition of *N*-centered radicals (promoted by boron catalysis, metalaphotoredox catalysis, or electron donor-acceptor (EDA) complex) to [1.1.1]propellane for the build-up of BCPAs has been established by limited individual groups (Scheme 1C, VII–IX) (Kim et al., 2020; Pickford et al., 2021; Shin et al., 2021). Nonetheless, the challenge of narrow substrate scopes and limited *N*-centered radical species remains unsolved. Considering the great potential of this valuable motif in synthetic and medicinal chemistry, sufficient attention from the scientific community should be paid to this promising motif.

Although several essential reviews focusing on constructing BCP derivatives have recently been published (He et al., 2020; Anderson et al., 2021), the advancement of radical chemistry in synthesizing BCPAs still lacks a concise review in this fast-growing and exciting field (Yu and Shi, 2022). In this review, we concisely summarize the fast-growing development of radical chemistry in the assembly of BCPAs motif, highlighting two different and powerful radical-involved strategies triggering the cleavage of the central bond of [1.1.1]propellane. The future direction concerning BCPAs is also discussed at the end of this review, which aims to provide some inspiration for the research of this promising project.

## Amination strategies to bicyclo[1.1.1]pentylamines

### Amination triggered by *C*-centered radicals

**Scheme 2:**
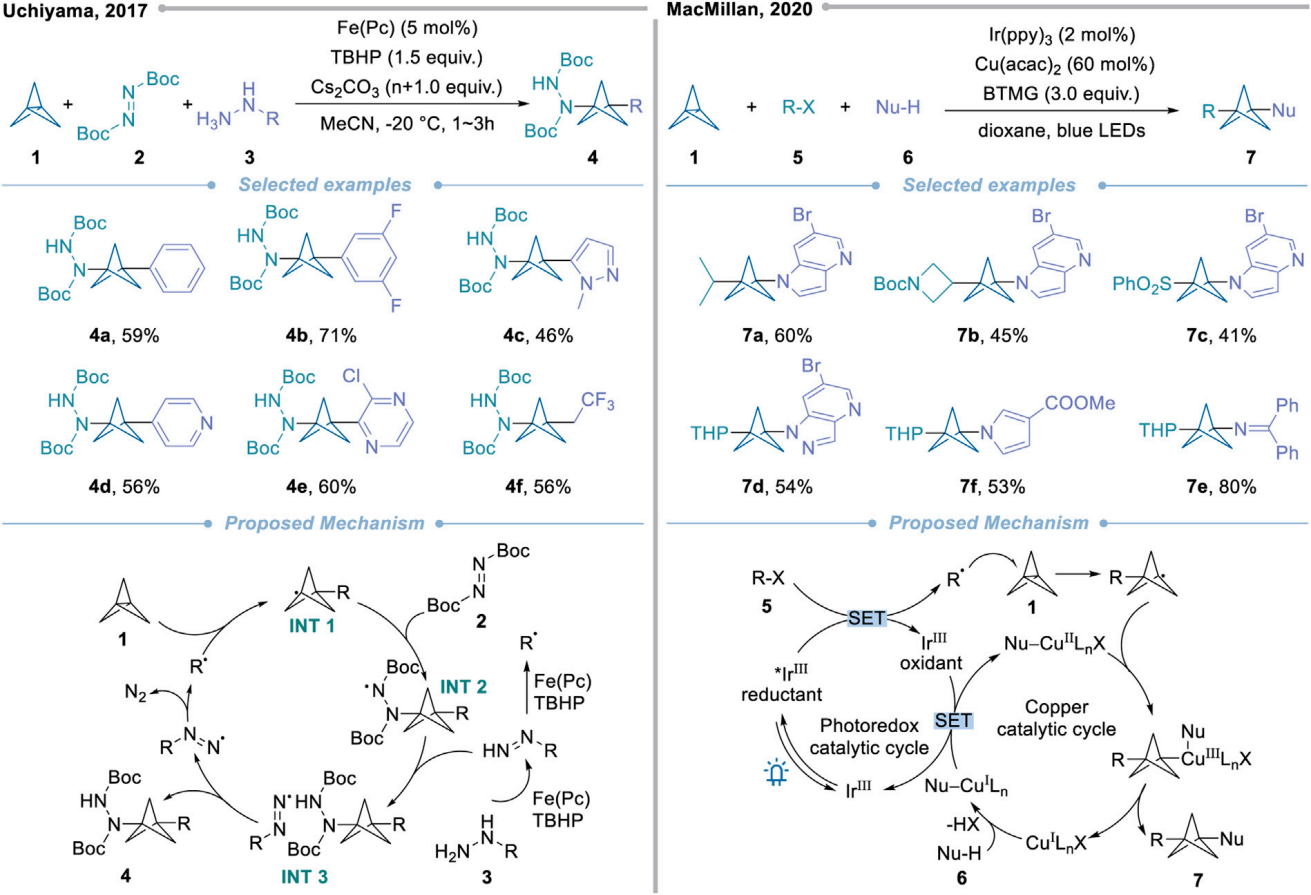
Approaches of *C*-centered radicals to valuable BCPAs.

In 2019, Uchiyama’s group developed the first radical multicomponent carboamination of [1.1.1]propellane to synthesize multi-functionalized BCPA derivatives (Scheme 2, left) (Kanazawa et al., 2017). With the aid of density functional theory (DFT) calculations, it was found that di-tert-butyl azodicarboxylate **2**, as the free radical receptor of 3-substituted BCP-radical (**INT 1**), can produce more stable free radical intermediates (**INT 2**). This chemical property of the substrate can avoid the self-polymerization of **1** over the reaction process and make the effective free radical chain reaction possible. After optimizing the conditions of free radical multicomponent reaction, the optimal conditions were determined with iron(II) phthalocyanine [Fe(Pc)] as a catalyst, tert-butyl hydroperoxide (TBHP) as oxidant, and Cs_2_CO_3_ as additive. Finally, under the condition of a tin-free/photoirradiation-free system, a series of novel multi-functionalized bicyclo[1.1.1]pentane derivatives were synthesized in one-pot operation with a yield of 38%–72%.

Although the above scheme proves the feasibility of the single-step carboamination multicomponent reaction *via* radical addition, the applicability of the reaction is still limited because only hydrazine is used as the free radical precursor. In 2019, McMillan and his research team made a breakthrough in this field (Scheme 2, right) (Zhang et al., 2020). Using various free radical precursors **5** and heteroatom nucleophiles **6**, they prepared a series of different functionalized dicyclopentanes with good yields through metal photo-oxidation-reduction catalytic reactions. Based on the importance of copper catalyst to the reaction system, the author first investigated copper salts and ligands and found that diketonate ligands such as acetylacetonate (acac) can effectively form the required three-component products, thus effectively avoiding the occurrence of free radical side reactions. Mechanistic investigation showed that the alkyl radical intermediates were generated under the catalytic cycle of the photocatalyst Ir(ppy)_3_. Subsequently, the nucleophile-ligated copper complex captured the BCP radical generated by radical addition. Eventually, the target product was obtained by reductive elimination.

### Amination triggered by *N*-centered radicals

**Scheme 3:**
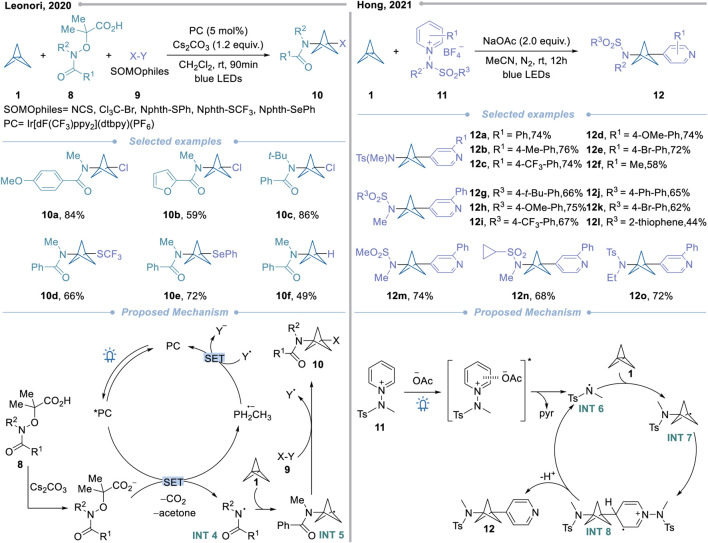
Approaches of *N*-centered radicals to construct valuable BCPAs.

Compared to *C*-centered radicals, the direct use of *N*-centered radicals to construct disubstituted BCPAs would be a more challenging path due to the susceptibility of nitrogen radicals to background reactions such as hydrogen extraction (Suarez et al., 2013; Xiong and Zhang, 2016; Kwon et al., 2022). In 2020, Leonori’s team reported the divergent strain-release amino-functionalization of [1.1.1]propellane with electrophilic nitrogen radicals for the first time (Scheme 3, left) (Kim et al., 2020). Based on the team’s previous research work, they hypothesized that under visible light excitation, the photocatalyst would be able to oxidize the carboxylic acid functional group of the free radical precursor **8**, triggering the extrusion of carbon dioxide and acetone and form the amide radical **INT 4**. Then, by using the electrophilic property of the species, the electron-rich **1** was successfully intercepted, the free radical strain release amination was realized, and the BCPA radical **INT5**. Finally, the species provides a variety of target components **10** through atom/group transfer reactions (S_H_2) with a series of SOMOphiles (X-Y) **9**. In terms of substrate adaptability, to meet the kinetic priority of the final atom/group transfer step over BCP radical oligomerization, they combined with density functional calculation to avoid side reactions. They found that six different pro-SOMOs were compatible, which supported the construction of various target products. This method dramatically expands the range of substituted BCPA obtained by free radical addition and may be further extended to other electrophilic free radicals.

Recently, the strategy of electron donor-acceptor (EDA) complexes has been widely explored to drive a new visible-light-induced conversion mode without an external photocatalyst (Rosokha and Kochi, 2008; Crisenza et al., 2020). In 2021, Hong’s team successfully provided BCPA through N-center free radicals based on this strategy (Scheme 3, right) (Shin et al., 2021). This strategy involves the photoactive formation of EDA complexes between *N*-aminopyridine salts **11** and acetate anions, thereby generating electrophilic amino radicals **INT6**. This radical cleaves the centrals bond and provides BCP radical intermediate **INT7**. After that, BCP radical **INT7** is ready to be added to the C4 position of another *N*-aminopyridine salt. Next, the resulting cationic radical species **INT8** undergoes deprotonation and cleavages N-N bond to get the final product **12** and amidyl radical **INT6**. This method directly introduces amide and pyridyl functions on the BCP core. In addition, it can also be extended to P and CF_3_ radicals, which have a good range of substrates.

**Scheme 4:**
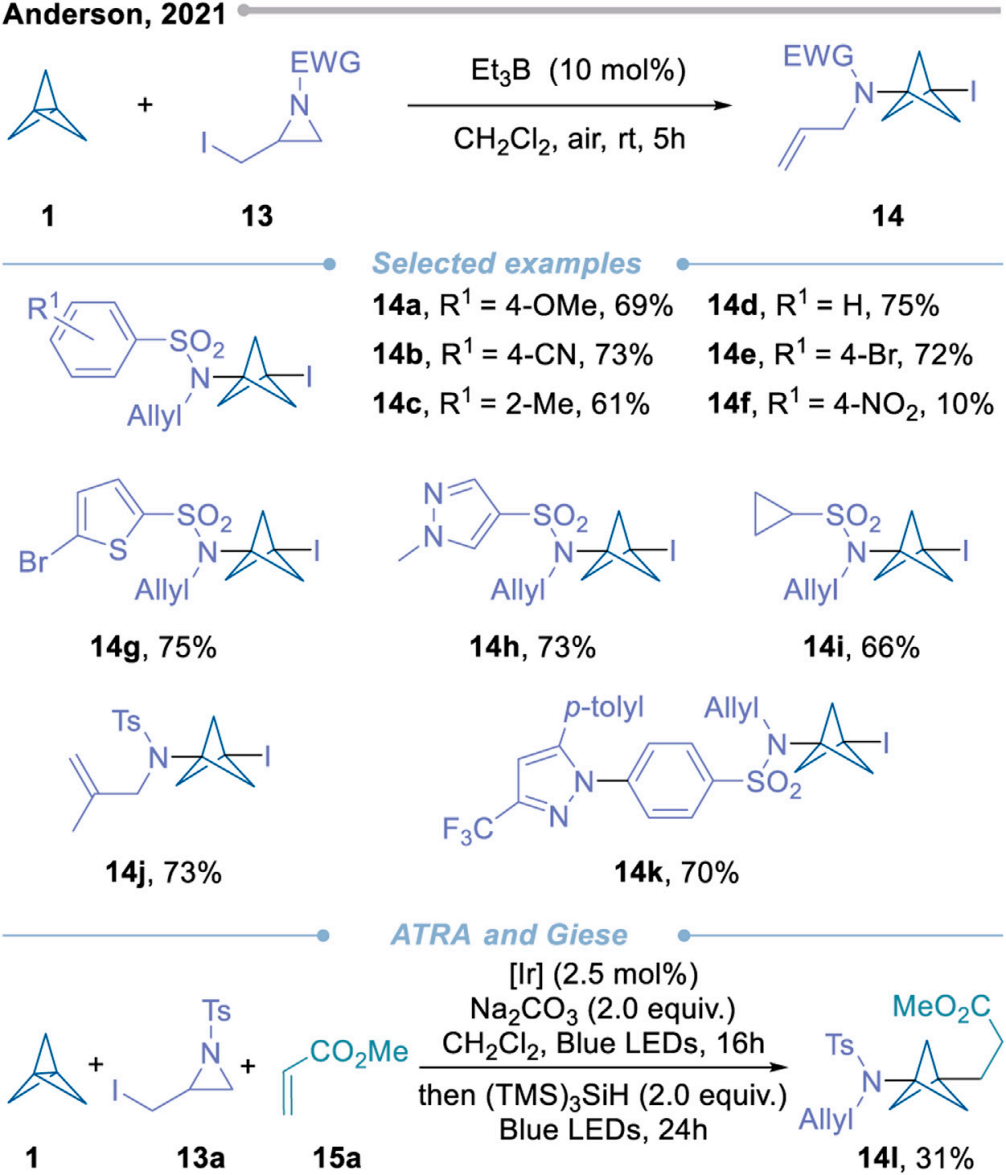
Approach of *N*-centered radical to construct valuable BCPAs.

In the same year, Anderson’s research group reported a twofold radical functionalization strategy, which provides a general and convenient new way to prepare 1,3-disubstituted iodo-BCPs (Scheme 4) (Pickford et al., 2021). In this method, *N*-centered radicals were obtained by fragmentation of α-iodoaziridines, which reacted with free radical receptor **1** to form a BCP radical, and then an iodine atom was extracted from the starting material of iodoazacyclopropane. These products can be further obtained by photoredox catalyzed Giese type reaction to obtain C-substituted BCPA. Interestingly, Anderson and his team succeeded in carrying out the ATRA/Giese process in a single reaction, and the overall yield of the transformation was consistent with the isolated steps.

## Conclusion

Due to its unique structural features and physicochemical profiles in medicinal and synthetic chemistry for introducing a three-dimensional cyclic moiety into small molecules or drug candidates, BCPAs scaffold, emerging as sp^3^-rich surrogates for aniline and its derivatives, have recently received increasing attention from the scientific community and industries. Overall, two different approaches for synthesizing BCPAs have been developed, mainly including adding *C*-centered or *N*-centered radicals to [1.1.1]propellane, respectively. Although fast-growing achievements in this field have been witnessed over the past years, compared with remarkable advancements in the construction of BCP frameworks, sufficient attention has not been paid to the construction of these valuable BCPAs frameworks, which is anticipated to be further explored soon. For instance, the current synthesis of BCPAs depends on limited radical species of *C*-centered or *N*-centered radicals; enriching the chemical tools of radical chemistry, such as flow chemistry or electrochemistry, could provide concise access to multifunctional BCPAs (Wegner et al., 2012; Elliott et al., 2014; Porta et al., 2016; Zhao and Xia, 2018; Donnelly and Baumann, 2021). Besides, discovering new reactivity with [1.1.1]propellane at the bridgehead position under a strain-release approach could offer an alternative solution for the amination functionalization of BCP molecules. Moreover, the asymmetric catalytic synthesis of optically pure BCPA derivatives has not been achieved; novel strategies and power catalysts are highly demanded to broaden the application range and explore the potential reactivity of [1.1.1]propellane (Meggers, 2015; Dilmac et al., 2017; Wong et al., 2019). Notably, the bio-evaluation of desirable BCPAs for new drug discovery and research goes far behind its synthetic chemistry (Bauer et al., 2021). Further medicinal research on those valuable compounds should soon be devoted to this field. We believe that further development of radical chemistry methods will significantly accelerate advances in synthesizing BCPAs and their application in drug discovery.
